# Arsenic trioxide sensitivity is associated with low level of glutathione in cancer cells

**DOI:** 10.1038/sj.bjc.6690766

**Published:** 1999-11

**Authors:** C-H Yang, M-L Kuo, J-C Chen, Y-C Chen

**Affiliations:** Department of Oncology, Graduate Institute of Medicine, Institute of Toxicology andDepartment of Laboratory Medicine, National Taiwan University Hospital and Medical Colleg, National Taiwan Universitye, Taipei, Taiwan

**Keywords:** arsenic trioxide, glutathione, buthionine sulphoximine

## Abstract

Arsenic trioxide (As_2_O_3_) is a novel anticancer agent, which has been found to induce remission in acute promyelocytic leukaemic patients following daily intravenous administration. The therapeutic value of As_2_O_3_ in other cancers is still largely unknown. Cytotoxic tests in a panel of cancer cell lines showed that bladder cancer, acute promyelocytic leukaemic and gastrointestinal cancer cells were the most sensitive to As_2_O_3_ among 17 cell lines tested. Cellular glutathione (GSH) system plays an important role in arsenic detoxification in mammalian cells. Cancer cells that were intrinsically sensitive to As_2_O_3_ contained lower levels of GSH, whereas resistant cancer cells contained higher levels of GSH. On the other hand, there was no association of glutathione-S-transferase-π or multidrug resistance-associated protein 1 levels with arsenic sensitivity in these cancer cells. Multidrug-resistant cancer cells that were cross-resistant to arsenic contained higher levels of GSH or multidrug-resistance-associated protein 1 than their drug-sensitive parental cells. Cancer cells become more sensitive to arsenic after depletion of cellular GSH with L-buthionine sulphoximine. We concluded that cellular GSH level is the most important determinant of arsenic sensitivity in cancer cells. Cellular GSH level and its modulation by buthionine sulphoximine should be considered in designing clinical trials using arsenic in solid tumours. © 1999 Cancer Research Campaign


*British Journal of Cancer* (1999) **81**(5), 796–799
© 1999 Cancer Research Campaign
Article no. bjoc.1999.0766

### Arsenic trioxide sensitivity is associated with low level of glutathione in cancer cells

**C-H Yang[1,2], M-L Kuo[3], J-C Chen[1] and Y-C Chen[4]**

[1]Department of Oncology, [2]Graduate Institute of Medicine, [3]Institute of Toxicology and [4]Department of Laboratory Medicine, National Taiwan University Hospital and Medical College, National Taiwan University, Taipei, Taiwan

**Summary** Arsenic trioxide ($As_2O_3$) is a novel anticancer agent, which has been found to induce remission in acute promyelocytic leukaemic patients following daily intravenous administration. The therapeutic value of $As_2O_3$ in other cancers is still largely unknown. Cytotoxic tests in a panel of cancer cell lines showed that bladder cancer, acute promyelocytic leukaemic and gastrointestinal cancer cells were the most sensitive to $As_2O_3$ among 17 cell lines tested. Cellular glutathione (GSH) system plays an important role in arsenic detoxification in mammalian cells. Cancer cells that were intrinsically sensitive to $As_2O_3$ contained lower levels of GSH, whereas resistant cancer cells contained higher levels of GSH. On the other hand, there was no association of glutathione-S-transferase-π or multidrug resistance-associated protein 1 levels with arsenic sensitivity in these cancer cells. Multidrug-resistant cancer cells that were cross-resistant to arsenic contained higher levels of GSH or multidrug-resistance-associated protein 1 than their drug-sensitive parental cells. Cancer cells become more sensitive to arsenic after depletion of cellular GSH with L-buthionine sulphoximine. We concluded that cellular GSH level is the most important determinant of arsenic sensitivity in cancer cells. Cellular GSH level and its modulation by buthionine sulphoximine should be considered in designing clinical trials using arsenic in solid tumours.

**Keywords:** arsenic trioxide; glutathione; buthionine sulphoximine

Arsenic trioxide ($As_2O_3$) is a novel anticancer agent. Daily intravenous infusion of 10 mg $As_2O_3$ induced complete remission in acute promyelocytic leukaemia patients who were refractory to conventional chemotherapy and/or all-trans retinoic acid (Shen et al, 1997; Soignet et al, 1998). The role of arsenic in the treatment of this unique form of leukaemia is still under investigation. Arsenic compounds have been tested in other haematological malignancies in vitro (Konig et al, 1997). It is not known at present whether these compounds will prove useful in the treatment of solid tumours. In order to investigate whether arsenic is as cytotoxic in acute promyelocytic leukaemia cells as in other solid tumour cells, we screened a panel of cancer cell lines with $As_2O_3$.

Multiple mechanisms of arsenic resistance in either bacteria or mammalian cells have been described in the literature. One of the most important arsenic detoxification mechanisms is the glutathione (GSH) system. Trivalent arsenic was shown to bind to GSH in a cell free system (Scott et al, 1993). Overexpression of glutathione-S-transferase-π (GST-π) (Lo et al, 1992), GSH (Huang et al, 1993) or multidrug resistance-associated protein (MRP1) (Cole et al, 1994) has been shown to confer arsenic resistance. To investigate the correlation of the GSH detoxification system with arsenic resistance, we examined GSH content, MRP1 and GST-π expression in a panel of cancer cells and in multidrug-resistant cancer cells that were cross-resistant to arsenic. L-buthionine sulphoximine (BSO) was used to modulate cellular GSH content and to enhance arsenic sensitivity of cancer cells.

*Received 15 February 1999*
*Revised 30 April 1999*
*Accepted 5 May 1999*

*Correspondence to:* C-H Yang, Department of Oncology, National Taiwan University Hospital, 7 Chung-Shan South Road, Taipei, Taiwan 10016

### MATERIALS AND METHODS

### Cell lines

Seventeen cell lines were examined. Three cell lines were developed from patients living in an arsenic-polluted area (blackfoot disease area). NTU-B1 was developed from a transitional bladder carcinoma patient (Yu et al, 1992). BFTC-905 was derived from a bladder papillary transitional cell carcinoma and BFTC-909 from a renal pelvis transitional cell carcinoma patient (Tzeng et al, 1996). BFTC-905 and BFTC-909 were obtained from the Culture Collection Research Center, Food Industry Research and Development Institute, Taiwan. NB4 cells were kindly provided by Dr H-C Hsu (Veterans General Hospital, Taipei, Taiwan). A2780, SW620, MCF7, A172, HepG2 were kindly provided by Dr KH Cowan (National Cancer Institute, Bethesda, MD, USA). TSGH8302 was kindly provided by Dr Y-S Chen (Institute of Biomedical Science, Academia Sinica, Taiwan). CL-1 cells were provided by Dr P-C Yang (National Taiwan University Hospital, Taiwan). NTU-B1/P14 is a cisplatin-resistant variant of NTU-B1 cells and was a gift from Dr Y-S Pu, MCF7/VP is a VP-16-resistant MCF7 cells (Schneider et al, 1994) and was a gift from Dr E Schneider (Wadworth, NY, USA). All other cell lines were purchased from American Tissue Culture Collection (Rockville, MD, USA).

### Cytotoxicity assay

Cells were distributed in 96-well culture plates. Various concentrations of $As_2O_3$ (Sigma, St Louis, MO, USA) were added to the cells growing at 37°C in Dulbecco's modified Eagle's medium (DMEM) containing 10% fetal calf serum in triplicate. After 96 h, the survival fraction was measured by the sulphorhodamine B

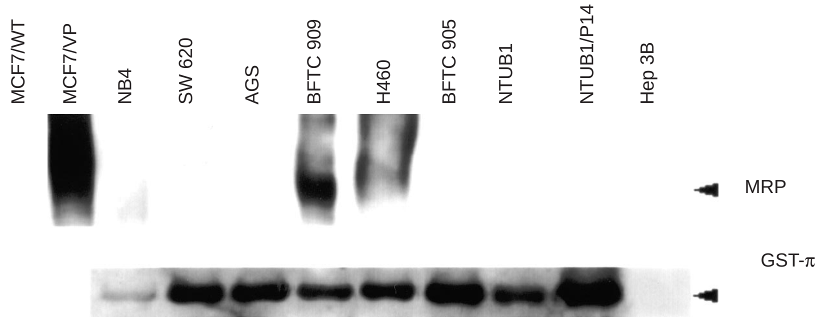

**Figure 1** Western blot of MRP (upper lane) and GST-π (lower lane) in cancer cells. MRP (190 kDa) was expressed in measurable amount only in MCF7/VP, BFTC909 and H460 cells. GST-π (26 kDa) was expressed in almost all cancer cells except for Hep3B cells method as described previously (Skehan et al, 1990).

### Cellular GSH content

Cellular GSH content was determined by Bioxytech GSH-400 colourimetric assay kit (Oxis International, Portland, OR, USA). Cells ($10^6$–$10^7$) were trypsinized, centrifuged and washed with phosphate-buffered solution. Cells were then re-suspended in 500 µl of ice-cold metaphosphoric acid. After homogenization, the solution was centrifuged at 3000 ***g***, 4°C for 10 min. The clear supernatant was collected at 4°C for further assay. Reagent R1 and sodium hydroxide were added to the solution. After incubation at 25°C for 10 min in the dark, the absorbance of the solution was measured at 400 nm. GSH concentrations in the solution were calculated from the absorbance. Cellular GSH content is expressed as µg of GSH $mg^{-1}$ of protein.

### Western blot of GST-π and MRP1

Total protein of the cells was separated by sodium dodecyl sulphate polyacrylamide gel electrophoresis (SDS-PAGE) and transferred to polyvinylidine difluoride (PVDF) membrane. The membrane was blocked in 5% skim milk in Tris-buffered saline, containing 0.15% Tween for 1 h before washing three times in the same solution containing 0.01% Tween. The membrane was then incubated with a 1:1000 dilution of polyclonal antibody against human GST-π (Medical and Biological Laboratory, Nagoya, Japan) for 1 h. Membrane was washed and incubated with HRP-conjugated secondary antibody. The immunolabelled protein was detected using a chemiluminescence kit (NEN Life Science, Boston, MA, USA).

Membrane protein was isolated from cancer cells. MRP1 levels were measured by Western blot using 1:200 dilution of monoclonal antibody MRPm6 (Sanbio, Uden, The Netherlands) as described previously (Flens et al, 1994). The immunolabelled protein was visualized with a chemiluminescence kit (NEN Life Science, Boston, MA, USA).

### RESULTS

### Cytotoxicity of $As_2O_3$ in cancer cells

Concentrations of $As_2O_3$ that inhibit 50% of cell growth ($IC_{50}$s) are listed in Table 1. Several bladder cancer (NTUB1, BFTC905, T24 and HTB-9) and gastrointestinal cancer cell lines (SW620 and AGS) were relatively sensitive to $As_2O_3$ in addition to acute promyelocytic leukaemic cells (NB4).

### GSH content in cancer cells

GSH contents in cancer cells are shown in Table 1. To correlate GSH contents to $IC_{50}$s of nine tested cell lines, Spearman's rho correlative coefficient was 0.661 ($P = 0.026$, one-tail). Five cell lines that were intrinsically sensitive to arsenic ($IC_{50}$s < 1.5 µM) all contained a low level of GSH (GSH < 10 µg $mg^{-1}$ protein), whereas four cell lines that were intrinsically resistant to arsenic ($IC_{50}$s > 1.5 µM) all contained a high level of GSH (GSH > 10 µg $mg^{-1}$ protein).

### GST-π protein expression in cancer cells

Overexpression of GST-π may facilitate conjugation of trivalent arsenic to GSH. GST-π protein expression in the cancer cells was measured by Western blot as shown in Figure 1. Arsenic-sensitive NB4 cells contained very low levels of GST-π protein. However, several arsenic-sensitive cells, such as BFTC905 and SW620 cells, expressed high levels of GST-π protein, whereas arsenic-resistant H460, Hep3B and BFTC909 cells expressed low levels of GST-π protein. There was no correlation of GST-π levels to $As_2O_3$ $IC_{50}$s in cancer cells. Multidrug-resistant NTU-B1/P14 cells overexpressed GST-π compared to their drug-sensitive parental NTU-B1 cells.

### MRP1 expression in cancer cells

MRP1 may facilitate export of conjugated GSH out of the cells (Rappa et al, 1997) and thus, may affect arsenic resistance in cancer cells. MRP1 expression in the membrane protein of cancer cells was measured by Western blot as shown in Figure 1. H460, BFTC909 and one multidrug-resistant MCF7/VP cell contained measurable levels of MRP1, whereas MRP1 expression was very low in other cancer cells. MRP1 seemed to confer resistance to arsenic; however, not all arsenic-resistant cancer cells expressed high levels of MRP1.

### Cross-resistance of arsenic in multidrug-resistant cancer cells

$IC_{50}$s of two multidrug-resistant cancer cells are listed in Table 1. Cisplatin-resistant NTU-B1/P14 was 5.5-fold resistant to arsenic. Etoposide-resistant MCF7/VP cells were 4.8-fold resistant to

© 1999 Cancer Research Campaign

**Table 1** Fifty per cent growth inhibitory concentrations ($IC_{50}$s) of arsenic trioxide on human cancer cells after 96 h of treatment

| Cell lines | Organ origin | $As_2O_3IC_{50}s^a$ | GSH content[b] |
|---|---|---|---|
| BFTC-905 | Bladder | 0.34 ± 0.03 | 6.03 ± 1.35 |
| NTU-B1 | Bladder | 0.47 ± 0.08 | 7.59 ± 1.16 |
| NTU-B1/P14 | Bladder | 2.59 ± 0.41 | 50.9 ± 10.9 |
| NB4 | Leukaemia | 0.64 ± 0.11 | 6.12 ± 0.96 |
| T24 | Bladder | 0.93 ± 0.20 | ND |
| A2780 | Ovary | 1.12 ± 0.33 | ND |
| SW620 | Colon | 1.16 ± 0.15 | 4.91 ± 0.17 |
| AGS | Stomach | 1.16 ± 0.20 | 7.30 ± 0.84 |
| HTB-9 | Bladder | 1.38 ± 0.04 | ND |
| MCF-7 | Breast | 2.08 ± 0.40 | 28.6 ± 2.3 |
| MCF7/VP | Breast | 9.89 ± 1.74 | 20.1 ± 0.8 |
| TSGH8302 | Cervix | 2.50 ± 0.69 | ND |
| BFTC-909 | Kidney | 2.84 ± 0.79 | 17.03 ± 2.88 |
| H460 | Lung | 3.27 ± 0.49 | 21.84 ± 2.89 |
| ES-2 | Ovary | 3.30 ± 1.36 | ND |
| A172 | Glioblastoma | 3.40 ± 0.40 | ND |
| CL-1 | Lung | 4.17 ± 0.50 | ND |
| Hep3B | Liver | 5.17 ± 1.02 | 17.02 ± 1.37 |
| HepG2 | Liver | 7.17 ± 1.20 | ND |
| Co-incubation with 10 μM BSO | | | |
| NTU-B1 | Bladder | 0.19 ± 0.04 | 2.28 ± 0.64 |
| NTU-B1/P14 | Bladder | 0.14 ± 0.01 | 14.20 ± 1.59 |
| MCF-7 | Breast | 0.40 ± 0.12 | 3.65 ± 0.23 |
| MCF7/VP | Breast | 0.20 ± 0.02 | 2.10 ± 0.12 |

Shown in the Table are the means and standard errors of at least three independent experiments. [a]μM, [b]μg GSH $mg^{-1}$ protein, ND: not done.

arsenic. Glutathione content of NTU-B1/P14 was 6.7-fold higher than that of NTU-B1 cells. On the other hand, there was no difference of GSH content between MCF7/WT and MCF7/VP cells. MCF7/VP expressed high levels of MRP1, which may account for its arsenic resistance, whereas NTU-B1/P14 expressed no measurable level of MRP1 (Figure 1).

### Modulation of GSH content in cancer cells by BSO

BSO is known to deplete cellular GSH via inhibition of γ-glutamylcysteine synthetase, which is required for GSH biosynthesis. NTU-B1, NTU-B1/P14, MCF7/WT and MCF7/VP cells were incubated with various concentrations of $As_2O_3$ and 10 μM of BSO for 4 days. Ten micromolars of BSO were not toxic to these cancer cells ($IC_{10}$s of BSO in NTU-B1, NTU-B1/P14, MCF7/WT and MCF7/VP cells were 37 μM, > 50 μM, 27 μM and 24 μM respectively). The representative cytotoxicity curves of NTU-B1 and NTU-B1/P14 cells in $As_2O_3$ with or without co-incubation with 10 μM of BSO are shown in Figure 2. $IC_{50}$s of $As_2O_3$ and GSH contents in BSO-treated treated GSH-depleted cells (drug-sensitive and -resistant NTU-B1 and MCF7/WT cells) are shown in Table 1. All four cancer cells became very sensitive to arsenic ($IC_{50}$s 0.1 μM to 0.4 μM) when GSH was depleted by BSO.

### DISCUSSION

Arsenic has been used widely for a long time in both Western and Chinese medicine. The definitive role of arsenic as an anticancer agent was not clear until a recent report for treatment of acute promyelocytic leukaemia (Shen et al, 1997). $As_2O_3$, given as a daily 10 mg intravenous infusion seemed to be an effective and tolerable regimen for refractory acute promyelocytic leukaemic patients.

Induction of partial differentiation or induction of apoptosis have been proposed as the primary mechanism of cytotoxicity of arsenic to acute promyelocytic leukaemic cells (Chen et al, 1997). Due to the unique mechanism of action of arsenic to cancer cells, the attempt to use arsenic in malignancies other than acute promyelocytic leukaemic patients is clearly warranted (Gallagher, 1998).

In this study, bladder cancer cells NTU-B1 and BFTC905 were most susceptible to $As_2O_3$. Apoptosis can be induced in NTU-B1 cells at 1 μM $As_2O_3$ (data not shown). $IC_{50}$s of the bladder cancer cell lines was substantially lower than reported in $As_2O_3$ peak plasma levels (4–6 μM) in patients (Shen et al, 1997). Thus, it is conceivable that $As_2O_3$ may be effective in the treatment of bladder cancer and other solid tumours that show similar sensitivity to arsenic.

Multiple mechanisms account for arsenic resistance in bacteria and mammalian cells. Trivalent arsenic was shown to directly react to reduced GSH in solution (Scott et al, 1993). The cellular

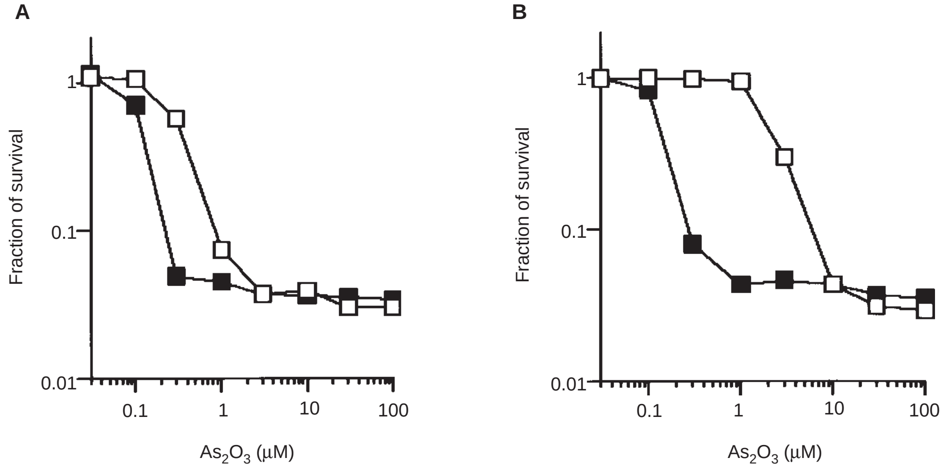

**Figure 2** Representative cytotoxicity curves of $As_2O_3$ in NTU-B1 cells (**A**) and NTU-B1/P14 cells (**B**). Cells were incubated in the presence (–■–) or absence (–□–) of 10 μM BSO

 © 1999 Cancer Research Campaign arsenic content was reduced by GSH pretreatment and increased in BSO-treated Chinese hamster ovary cells (Huang et al, 1993). It is conceivable that cellular GSH content affects sensitivity of cancer cells to $As_2O_3$. In this study, we have shown clearly that cancer cells that contained low levels of GSH were all sensitive to arsenic exposure, whereas resistant cancer cells such as lung and liver cancer cells contained the highest amounts of GSH. Furthermore, sensitivity of NTU-B1 and MCF7 cells to arsenic can be increased when GSH was depleted by pretreatment with 10 μM BSO. Cisplatin-resistant NTU-B1/P14 cells were cross-resistant to arsenic, the GSH content of these cells was higher than in steady-state parental cells. When GSH was depleted by BSO, resistant cells became sensitive to arsenic treatment. BSO may also enhance arsenic toxicity in wild-type MCF7 cells and multidrug-resistant and MCF7/VP cells. When cellular GSH was depleted, all drug-sensitive and multidrug-resistant cancer cells became very sensitive to arsenic.

GST-π overexpression was noted in a Chinese hamster ovary cells resistant to $As_2O_3$ (CHO/SA7) (Lo et al, 1992). It is conceivable that GST-π protein levels may also affect intrinsic sensitivity to arsenic. The GST-π level seems to play very little role, however, in the determination of arsenic sensitivity of cancer cells in this study.

MRP1-transfected HeLa cells were resistant to several heavy metal anions, including trivalent arsenic (Cole et al, 1994). In this study, multidrug-resistant cancer cells such as MCF7/VP and NTU-B1/P14 cells were cross-resistant to $As_2O_3$. MRP1 was overexpressed in several arsenic-resistant cancer cells such as BFTC909, H460 and MCF7/VP cells. However, MRP1 was not expressed in meaningful amounts in intrinsically resistant MCF7, HepB3 cells or acquired resistant NTU-B1/P14 cells. We demonstrated complete reversal of arsenic resistance in MRP1-overexpressing MCF7/VP cells when GSH was depleted by BSO. The result suggests that MRP1 overexpression may not protect cancer cells from arsenic toxicity when GSH was depleted. Overexpression of MRP1 may contribute to arsenic resistance, but MRP1 expression is not the main determinant of arsenic sensitivity in cancer cells.

Use of $As_2O_3$ in solid tumour clinical trials is clearly warranted. Our study suggests that GSH content in tumour cells may be the main determinant of arsenic sensitivity. Attempts should be made to measure tumour GSH content and correlate to arsenic response in clinical trials. BSO was used to deplete GSH and enhance chemosensitivity of alkylating agents (Bailey et al, 1994). The peak plasma level of BSO in patients (4–6 mM) was much higher than levels needed to enhance arsenic toxicity in arsenic resistant cancer cells (10 μM). Therefore, adding BSO to arsenic treatment may potentially be useful to reverse acquired arsenic resistance in acute promyelocytic leukaemic patients or to treat tumours that are intrinsically resistant to arsenic.

In conclusion, GSH content correlates well with arsenic resistance in cancer cells. Depletion of cellular GSH by BSO enhanced arsenic toxicity in both arsenic-sensitive and -resistant cancer cells. Further animal studies and human trials evaluating arsenic as an anticancer drug are warranted. Our study suggests that $As_2O_3$ should be tested in solid cancers, especially patients with bladder and gastrointestinal cancer. This study suggests that measurement and modulation of cellular GSH content in cancer cells should be deployed in designing future clinical trials.

### ACKNOWLEDGEMENTS

The authors thank Miss Tsui-Ying Wang, En-Tsy Chan and Mei-Chung Tu for technical assistance and Dr Ronald Freund for editing this manuscript. Supported by National Science Council, Taiwan Grant NSC 87–2314-B-002–079 and the National Taiwan University Hospital grant NTUHS871002

### REFERENCES

Bailey HH, Mulcahy RT, Tutsch KD, Arzoomanian RZ, Alberti D, Tombes MB, Wilding G, Pomplun M and Spriggs DR (1994) Phase I clinical trial of intravenous L-buthionine sulfoximine and melphalan: an attempt at modulation of glutathione. *J Clin Oncol* **12**: 194–205

Cole SP, Sparks KE, Fraser K, Loe DW, Grant CE, Wilson GM and Deeley RG (1994) Pharmacological characterization of multidrug resistant MRP-transfected human tumor cells. *Cancer Res* **54**: 5902–5910

Chen GQ, Shi XG, Tang W, Xiong SM, Zhu J, Cai X, Han ZG, Ni JH, Shi GY, Jia PM, Liu MM, He KL, Niu C, Ma J, Zhang P, Zhang TD, Paul P, Naoe T, Kitamura K, Miller W, Waxman S, Wang ZY, de The H, Chen SJ and Chen Z (1997) Use of arsenic trioxide ($As_2O_3$) in the treatment of acute promyelocytic leukemia (APL): I. $As_2O_3$ exerts dose-dependent dual effects on APL cells. *Blood* **89**: 3345–3353

Flens MJ, Izquierdo MA, Scheffer GL, Fritz JM, Meijer CJ, Scheper RJ and Zaman GJ (1994) Immunochemical detection of the multidrug resistance-associated protein MRP in human multidrug-resistant tumor cells by monoclonal antibodies. *Cancer Res* **54**: 4557–4563

Gallagher RE (1998) Arsenic: new life for an old potion. *N Engl J Med* **339**: 1389–1391

Huang H, Huang CF, Wu DR, Jinn CM and Jan KY (1993) Glutathione as a cellular defence against arsenite toxicity in cultured Chinese hamster ovary cells. *Toxicology* **79**: 195–204

Konig A, Wrazel L, Warrell RP Jr., Rivi R, Pandolfi PP, Jakubowski A and Gabrilove JL (1997) Comparative activity of melarsoprol and arsenic trioxide in chronic B-cell leukemia lines. *Blood* **90**: 562–570

Lo JF, Wang HF, Tam MF and Lee TC (1992) Glutathione S-transferase pi in an arsenic-resistant Chinese hamster ovary cell line. *Biochem J* **288**: 977–982

Rappa G, Lorico A, Flavell RA and Sartorelli AC (1997) Evidence that the multidrug resistance protein (MRP) functions as a co-transporter of glutathione and natural product toxins. *Cancer Res* **57**: 5232–5237

Schneider E, Horton JK, Yang CH, Nakagawa M and Cowan KH (1994) Multidrug resistance-associated protein gene overexpression and reduced drug sensitivity of topoisomerase II in a human breast carcinoma MCF7 cell line selected for etoposide resistance. *Cancer Res* **54**: 152–158

Scott N, Hatlelid KM, MacKenzie NE and Carter DE (1993) Reactions of arsenic(III) and arsenic(V) species with glutathione. *Chem Res Toxicol* **6**: 102–106

Shen ZX, Chen GQ, Ni JH, Li XS, Xiong SM, Qiu QY, Zhu J, Tang W, Sun GL, Yang KQ, Chen Y, Zhou L, Fang ZW, Wang YT, Ma J, Zhang P, Zhang TD, Chen SJ, Chen Z and Wang ZY (1997) Use of arsenic trioxide ($As_2O_3$) in the treatment of acute promyelocytic leukemia (APL): II. Clinical efficacy and pharmacokinetics in relapsed patients. *Blood* **89**: 3354–3360

Skehan P, Storeng R, Scudiero D, Monks A, McMahon J, Vistica D, Warren JT, Bokesch H, Kenney S and Boyd MR (1990) New colorimetric cytotoxicity assay for anticancer-drug screening. *J Natl Cancer Inst* **82**: 1107–1112

Soignet SL, Maslak P, Wang ZG, Jhanwar S, Calleja E, Dardashti LJ, Corso D, DeBlasio A, Gabrilove J, Scheinberg DA, Pandolfi PP and Warrell RP Jr (1998) Complete remission after treatment of acute promyelocytic leukemia with arsenic trioxide, *N Engl J Med* **339**: 1341–1348

Tzeng CC, Liu HS, Li C, Jin YT, Chen RM, Yang WH and Lin JS (1996) Characterization of two urothelium cancer cell lines derived from a blackfoot disease endemic area in Taiwan. *Anticancer Res* **16**: 1797–1804

Wang HF and Lee TC (1993) Glutathione S-transferase pi facilitates the excretion of arsenic from arsenic-resistant Chinese hamster ovary cells. *Biochem & Biophys Res Communication* **192**: 1093–1099

Yu HJ, Tsai TC, Hsieh TS and Chiu TY (1992) Characterization of a newly established human bladder carcinoma cell line, NTUB1. *J Formosan Med Ass* **91**: 608–613

© 1999 Cancer Research Campaign 
